# Molecular Correlates of Early Onset of Diabetic Cardiomyopathy: Possible Therapeutic Targets

**DOI:** 10.1155/2022/9014155

**Published:** 2022-04-14

**Authors:** Dongjuan Wang, Kun Liu, Jinyan Zhong, Xin Li, Jie Zhang, Gongxin Wang, Ni Li, Tianwen Li, Harvey Davis, Ibrahim El-gaby, Guoliang Hao, Honghua Ye, Dan Li

**Affiliations:** ^1^Department of Cardiology, HwaMei Hospital, University of Chinese Academy of Sciences, Ningbo, Zhejiang 315000, China; ^2^Burdon Sanderson Cardiac Science Centre and BHF Centre of Research Excellence, Department of Physiology, Anatomy and Genetics, University of Oxford, Oxford OX1 3PT, UK; ^3^Department of Endocrinology, HwaMei Hospital, University of Chinese Academy of Sciences, Ningbo, Zhejiang 315000, China; ^4^Henan SCOPE Research Institute of Electrophysiology Co. Ltd., Kaifeng 475000, China; ^5^Ningbo Institute of Life and Health Industry, University of Chinese Academy of Sciences, Ningbo, Zhejiang 315000, China; ^6^Department of Neuroscience, Physiology and Pharmacology, University College London, London WC1E 6BT, UK

## Abstract

Diabetes mellitus (DM) is associated with mitochondrial dysfunction and oxidative stress that can lead to diabetic cardiomyopathy (DCM), which can often remain undetected until late stages of the disease. However, myocardial injury occurs before the onset of measurable cardiac dysfunction, although its molecular correlates are poorly understood. In this study, we made a DM rat induced by a high-fat diet combined with low and high doses of streptozotocin (STZ) to emulate pre and early DCM. RNA-sequencing analysis of ventricular tissue revealed a differential transcriptome profile and abnormal activation of pathways involved in fatty acid metabolism, oxidative phosphorylation, cardiac structure and function, insulin resistance, calcium signalling, apoptosis, and TNF signalling. Moreover, using high glucose-treated human induced pluripotent stem cell-derived cardiomyocytes (iPSC-CM), we recapitulated the cardiac cellular phenotype of DM and identified several molecular correlates that may promote the development of DCM. In conclusion, we have developed an experimental framework to target pathways underlying the progression of DCM.

## 1. Introduction

Diabetes mellitus (DM) is a metabolic disorder that leads to chronic impairment of the cardiovascular system, kidneys, peripheral nervous system, and retina [1]. Accumulating evidence has demonstrated that DM (particularly type 2 diabetes, T2DM) adversely affects patient long-term survival [2]. In particular, T2DM is associated with a higher risk of cardiovascular disease-related death [3] that is coupled to diabetic cardiomyopathy (DCM), which is independent of hypertension, coronary artery disease, or valvular heart disease [4]. DCM is usually clinically asymptomatic can often remain undetected until late stages of the disease, although cardiac functional abnormalities is clearly present during the early stages of the disease.

Mitochondria supply most of the energy that is essential for the heartbeat and control basic cellular functions, including Ca^2+^ signalling homeostasis, ROS generation, and apoptotic cell death regulation [5]. In T2DM, both basic science and clinical studies suggest that cardiac impairment occurs as a result of dysregulated glucose and lipid metabolism, which leads to increased oxidative stress and the activation of multiple signal pathways resulting in myocardial injury [6]. High levels of reactive oxygen species (ROS) uncouple the mitochondria electron transport chain (ETC), thereby reducing mitochondrial ATP production. This eventually triggers mitochondrial permeability transition pore (mPTP) opening and cardiomyocyte death [7]. Although measures of cardiac performance can be used to assess subclinical cardiac abnormalities, myocardial injury occurs early and before the onset of measurable cardiac dysfunction. The extent to which diabetes status may confer the risk of myocardial injury remains unclear.

The present study was designed to characterize the underlying molecular correlates associated with cardiac abnormalities in a rat model of DM. We used whole transcriptome analysis with total RNA sequencing (RNA-Seq) to capture the effect of changes in gene expression caused by DM. To test whether these targets could be recapitulated in an analogue model of cardiac DM, we used high glucose-treated human induced pluripotent stem cell- (hiPSC-) derived cardiomyocytes to explore potential therapeutic target genes that may promote the development of DCM.

## 2. Materials and Methods

### 2.1. Rat DM Model Experiments

#### 2.1.1. Animal Preparation

Male Sprague–Dawley rats (250–300 g) were randomly assigned to 3 groups: control group (Con, *n* = 10), low dose group (Low, *n* = 15), and high dose group (High, *n* = 15). The control group was fed with a normal chow diet consisting of 12% fat, 66% carbohydrates, and 22% protein, and the other two groups were fed with a high-fat diet consisting of 60% fat, 5% carbohydrates, and 34% protein, according to the protocol of Mansor et al. [8]. After 2 weeks, the low and high groups received a single intraperitoneal injection of STZ (Sigma, Low: 35 mg/kg; High: 50 mg/kg) [9]. Fasting blood glucose levels were tested once a week. All experiments were conducted under the National Institutes of Health Guidelines on the Use of Laboratory Animal and were approved by the University of Chinese Academy of Sciences on Animal Care.

Nine weeks after STZ injection, rats fasted overnight and blood glucose, insulin, triglyceride (TG), total cholesterol (TC), low-density lipoprotein cholesterol (LDL-C), lactate dehydrogenase (LDH), and free fatty acid (FFA) were measured. An oral glucose tolerance test (OGTT) was performed as described previously [8]. The homeostasis model assessment estimated insulin resistance (HOMA-IR) was calculated as (fasting serum glucose × fasting serum insulin/22.5 [10]. The cardiac weight index was used to evaluate the hypertrophic response, which was calculated as the heart weight (HW, g)/body weight (BW, g) as previously described [11].

#### 2.1.2. Real-Time RT-PCR

Hearts from each group were isolated, and total mRNA was extracted with Trizol reagent (Invitrogen, USA). PrimeScript RT Master Mix kit (Takara Biotechnoly (Dalian) Co., China) was used to conduct reverse transcription, then, Fast SYBR Green Master Mix (Applied Biosystems, USA) was used to perform real-time RT-PCR (Applied Biosystems, USA). RT-PCR was carried out in a total volume of 20 *μ*l reaction mixture according to the manufacture's protocol. Amplifications were carried out as follows: 95°C for 3 min, 40 cycles of 95°C for 15 s, and 60°C for 45 s. Primers used are listed in Supplementary Table 1. Gene expression was normalized to GAPDH that was used as an internal control. Fold changes in gene expression were measured using the 2^-*ΔΔ*CT^ method.

#### 2.1.3. RNA-Seq and Bioinformatics

RNA-Seq was based on previous rat studies [12, 13]. Here, total RNA was extracted from freshly isolated rat left ventricular tissue (4 samples per group) by using the RNeasy Kit from QIAGEN (Qiagen, Hilden, Germany) with DNase I treatment used according to the manufacturer's protocol. RNA quality and concentration were tested with Bioanalyzer 2100 (Agilent, CA, USA). All samples fulfilled the criteria of RNA integrity number > 7 and concentration > 50 ng/*μ*l. Sequencing libraries were constructed in Beijing Genomics Institute (Beijing, China) using a modified protocol similar to the TrueSeq Stranded Total RNA with RiboZero Kit (Illumina Inc.). RNA sequence was performed on BGISEQ-500 platform.

SOAPnuke (v1.5.2) was used to filter reads and generate FASTQ format. Clean reads were aligned to the reference rat genome using the Bowtie2 (v2.2.5) with default parameters and calculated gene expression level with RSEM (v1.2.12). The differentially expressed genes (DEGs) were identified with more than double fold change, and the corrected *P* value is less than or equal to 0.05. In gene ontology (GO) analysis and pathway analysis, DEGs were classified according to official classification with the GO or Kyoto Encyclopedia of Genes and Genomes (KEGG) annotation results, and Phyper (a function of R) was performed on GO and pathway functional enrichment. The false discovery rate (FDR) was calculated for each *P* value with FDR ≤ 0.05 defined as significantly enriched. Protein-protein interaction results obtained from STRING database (v11.5) with default settings. Cytoscape software (v3.8.2) was applied to visualize the protein interaction relationship network and analyze hub genes.

#### 2.1.4. Histological Analysis

Hematoxylin–eosin (HE) staining and Masson's trichrome staining were conducted as previously described [14, 15]. Briefly, the excised heart tissues were embedded in paraffin, cut into 5 *μ*m thick serial sections, and then stained with HE or Masson's trichrome. Masson's trichrome was used to analyze the collagen fraction in the myocardium with cardiomyocytes stained red and collagen fibers stained blue. The percentage of the total area covered by collagen was analyzed with Image-Pro Plus software version 6.0, which calculated as follows: collagen area/view area × 100.

#### 2.1.5. DHE Staining

Dihydroethidine (DHE) staining was used to measure the intracellular ROS according to the oxidative fluorescent microtopography, as described recently [16]. Frozen left ventricular cardiac sections from rats were stained with 10 *μ*mol/L DHE in a dark and humidified chamber for 30 minutes at 37°C. Omission of DHE was used as a negative control. DHE staining was visualized under a confocal microscope (Olympus FV 1000, Tokyo, Japan), and images were analyzed with Image-Pro Plus software version 6.0. The mean fluorescence intensity of each section was calculated, and the total section emission signals per field were averaged for data analysis.

#### 2.1.6. Caspase-3 Expression

Caspase-3 expression in heart was measured in the heart via immunofluorescence staining. Briefly, the isolated heart tissues were embedded in a paraffin and cut into 5 *μ*m thick serial sections. After dehydrating, heat-induced antigen retrieval was performed by boiling the sections in citrate buffer, pH 6.0, in a microwave oven. To block endogenous peroxidase activity, sections were incubated in a solution of 3% H_2_O_2_ for 20 min at room temperature. Then, caspase-3 antibody (1:200; Tocris Bioscience) was incubated overnight at 4°C, followed by detection with corresponding fluorescent secondary antibodies (Santa Cruz Biotechnology, CA) for 1 h at 37°C. Nuclei were counterstained with DAPI. Samples were examined by a confocal microscope (Olympus FV 1000).

#### 2.1.7. TUNEL Assay

Terminal deoxy-nucleotidyl transferase-mediated dUTP nick end-labeling (TUNEL, in green) assay was conducted to detect myocardial apoptosis using an In Situ Cell Death Detection Kit (Roche, Germany) according to the manufacturer's instructions. The index of apoptosis was expressed as the proportion of TUNEL positive nuclei to the total number of nuclei (DAPI, in blue) in percentage.

#### 2.1.8. Optical Mapping of Ex Vivo Cardiac Preparations

The rats were euthanized (isoflurane; 1 ml/kg) and heparinized (120 IU). Hearts were excised and retrogradely perfused via the aorta with KH solution (in mmol/L: 119 NaCl, 4 KCl, 1.8 CaCl_2_, 1 MgCl_2_, 1.2 NaH_2_PO_4_, 25 NaHCO_3_, and 10 glucose; 95% O2/5% CO_2_) at 10 mL/min and 37°C. Dye loading was transfused by preperfusion with pluronic F127 (20% *w*/*v* in DMSO). Rhod2-AM (1 mg/ml) was perfused for 25 minutes at 37°C to perform Ca^2+^ measurements. The heart was illuminated by 530 ± 25 nm LED (MappingLab). The fluorescence light was bandpass filtered (wavelengths 511–551 nm) to minimize stray excitation light reaching the dyes. The emitted fluorescence signal was filtered with a 590 nm bandpass filter (bandwidth 35 nm), then imaged by a CMOS camera (OMS-PCIE-2002, MappingLab). Digital images (150 × 150 pixels) were gathered at a sampling rate of 0.8 kHz from a 2.2 × 2.2 mm field of view.

Optical mapping data were analyzed using a commercially available software (OMapScope5.7.8, MappingLab). Optical signals were spatially aligned and processed using a Gaussian spatial filter (3 × 3 pixels). Local activation time was assigned based on the maximum departure velocity of the Vm upstroke. Time to peak was defined as the time from initiation of Ca^2+^ to peak fluorescence. CTD90 was calculated.

### 2.2. Human Induced Pluripotent Stem Cell-Derived Cardiomyocyte Experiment

#### 2.2.1. hiPSC-CM Differentiation and Maturation

Healthy hiPSC lines SFC-854 and OX1-19 were cultured in mTeSR medium on Matrigel-coated plates and were dissociated using ReLeSR at 90-100% confluency. After 3 passages, the cells were then transferred onto Matrigel–coated 12 well plates for differentiation. The hiPSC-CM differentiation was carried out according to the procedure described in a previous report [17]. In brief, cells were cultured and expanded to 90% cell confluence and then treated for 2 days with 6 *μ*mol/L CHIR99201 in differentiation medium (RPMI medium supplemented with 1% B-27 supplement minus insulin). On day 3, cells were treated with 2.5 mmol/L Wnt-C59 to inhibit the Wnt signalling pathway. The purification medium was applied on day 11 and 13 by changing of the medium to no-glucose RPMI with 1% B27 minus insulin and 5 mmol/L sodium lactate. hiPSC-CM maturation was initiated from day 16 to day 20 with maturation medium (MM): DMEM containing 5 mmol/L glucose supplemented with 0.4 mmol/L oleic acid conjugated to BSA, 50 nmol/L insulin, 10% fetal calf serum inactive, 1% glutamine, and 1% penicillin–streptomycin (P/S). To achieve a high glucose environment, hiPSC-CM was treated with 5.5, 11, and 25 mmol/L glucose for 2 days.

#### 2.2.2. Cell Area and Nuclear Area Measurement

Cell size and nuclear size were measured using ImageJ (v1.53n, NIH, US). iPSCs-CMs were dissociated and replated in fluorodish, cultured in maturation medium for 2 days, and incubated with 5.5, 11, and 25 mmol/L glucose for 48 hours. Cells were fixed with 4% formaldehyde, permeabilized with 0.1% Triton, blocked with 6% donkey serum, and then stained with antibodies against the cytoskeleton protein troponin-T (ab45932) and *α*-actinin (sigmaA7811), and Hoechst for nucleus. Immunofluorescence images of the same scale were taken using a Leica confocal microscope (Leica TCS SP5) with a 60× oil immersion objective, which were then loaded in ImageJ for area measurement. For each image, all cells were manually selected with the freehand selection tool of ImageJ for the measurement of cell size, and nuclei were automatically selected by thresholding the images of the channel of nuclei.

#### 2.2.3. Glucose Uptake

hiPSC-CMs were seeded on 96-well plates (3.5 × 10^4^ cells/well), then, cells were treated with 5.5, 11, or 25 mmol/L glucose for 2 days. Glucose uptake of 2-deoxyglucose (2DG) was measured in hiPSC-CMs using a glucose uptake Glo^TM^ assay kit (Promega, J1342), and luminescence intensity (Relative light unit, RLU) was measured following the manufacturer's instruction.

#### 2.2.4. Production of Reactive Oxygen Species (ROS)

hiPSC-CMs were seeded in 96-well plates (3.5 × 10^4^ cells/well) and treated with 5.5, 11, or 25 mmol/L glucose for 2 days. ROS in live hiPSC-CMs were quantitatively assessed using a cellular ROS assay kit (ab113851, Abcam) according to the manufacturers' instructions.

#### 2.2.5. ATP Assay of Cell Viability

The amount of ATP in cells correlated with cell viability was determined by CellTiter-Glo 2.0 Cell Viability Assay (Promega, USA, G9242) according to the manufacturer's protocol. hiPSC-CMs were seeded on 96-well plates (3.5 × 10^4^ cells/well), incubated with 5.5, 11, or 25 mmol/L glucose for 2 days.

#### 2.2.6. Intracellular Free Calcium Concentration Measurement

Intracellular free calcium concentration ([Ca^2+^]_i_) using Fura-2-acetoxymethyl ester (Fura-2/AM) was performed as described earlier with slight modification [18]. Briefly, cultured hiPSC-CMs were incubated in 5 *μ*mol/L Fura-2/AM for 30 min at 37°C. Loaded hiPSC-CMs were imaged with a QICLICK digital CCD camera (Photometrics) connected to an OptoLED fluorescence imaging system housed on an inverted Nikon microscope equipped with a 40× oil immersion objective. The evoked [Ca^2+^]_i_ transient was evaluated by 30-second exposure to 1 Hz field stimulation or FCCP (carbonylcyanide-p-trifluoromethoxyphenylhydrazone, 3 *μ*mol/L). The ratio of the fluorescence intensity (R) emitted at 510 nm obtained by alternately exciting at 340 and 380 nm at each time interval is used to estimate the changes in intracellular free Ca^2+^ concentration.

#### 2.2.7. Detection of Mitochondrial Calcium

To measure mitochondrial Ca^2+^ ([Ca^2+^]_m_), hiPSC-CMs were loaded with 5 *μ*mol/LRhod-2/AM for 25 min at 37°C. Rhod-2 has a net positive charge, which promotes preferential sequestration in the mitochondria due to potential-driven uptake. FCCP-induced **[**Ca^2+^]_m_ release was assessed by the decline in Rhod-2/AM fluorescence intensity.

#### 2.2.8. Mitochondrial Membrane Potential Measurement

Mitochondrial membrane potential (MMP) change was assessed in hiPSC-CMs using the lipophilic cationic probe 5,5′,6,6′-tetrachloro-1,1′,3,3′-tetraethylbenzimidazol-carbocyanine iodide (JC-1,thermofisher, T3168) following the manufacturer's protocol. hiPSC-CMs were cultured at a density of 3.5 × 10^5^ cells/FluoroDish for 2 days and then treated with 5.5, 11, or 25 mmol/L glucose for a further 2 days. Cells were incubated with 2 *μ*mol/L JC-1 dye for 30 min at 37°C. The green and red fluorescences were measured using an Airyscan confocal microscope (ZEISS 880). The ratio of red to green mean fluorescence intensity was quantified using ImageJ.

### 2.3. Statistical Analysis

Statistical analyses were performed using Prism version 9 software (GraphPad Software, La Jolla, CA). Unpaired *t*-test or one-way analysis of variance was used to determine differences between the control and two treatment groups. The values are presented as the means ± SE. Statistically significant difference was set at *P* < 0.05.

## 3. Results

### 3.1. Assessment of Cardiac Parameters and Glucose Handling across Experimental Groups

**Figure 1:**
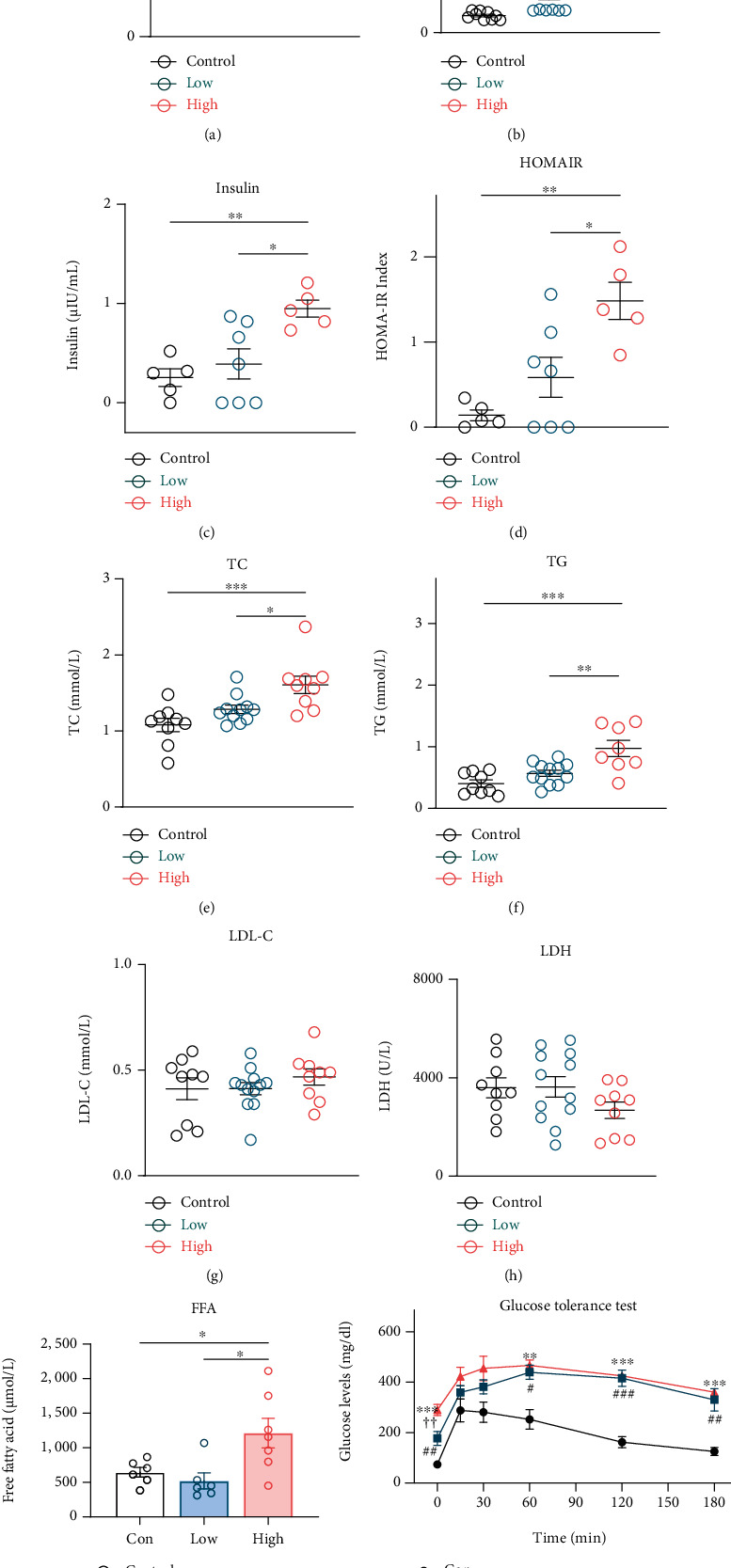
Basic parameters of rats in different experimental groups. (a) HR in the three groups. Serum levels of GLU (b), insulin (c), TC (e), TG (f), LDL-C (g), LDH (h), and FFA (i) in the three groups. (d) Assessment of insulin resistance in the three groups. (j) Mean plasma glucose concentration in response to an oral glucose challenge. Data are expressed as mean ± SE. ^∗^*P* < 0.05, ^∗∗^*P* < 0.01, ^∗∗∗^*P* < 0.001, ^∗∗∗∗^*P* < 0.0001. For (j): ^∗∗^*P* < 0.01, ^∗∗∗^*P* < 0.001, high STZ treated group compared with control; ^#^*P* < 0.05, ^##^*P* < 0.01, ^###^*P* < 0.001, low STZ group compared with control; ^††^*P* < 0.01, low STZ compare with high STZ treated group. Con: control group; Low: low STZ group; High: high STZ group; HR: heart rate; GLU: glucose; TC: total cholesterol; TG: triglyceride; LDL-C: low-density lipoprotein cholesterol; LDH: lactate dehydrogenase; FFA: free fatty acid. *n* = 9 − 12 per group.

After 9 weeks of STZ treatment, the heart rate did not differ among groups (Figure 1(a)). Rats from the high STZ group exhibited significant higher serum levels of glucose, insulin, HOMA-IR, TC, TG, and FFA compared with control. In addition, glucose, insulin, HOMA-IR, TG, and FFA in the high STZ group displayed a significant difference relative to the low group, and the glucose level in the low STZ was enhanced when compared to the control (Figures 1(b)–1(f) and 1(i)). Furthermore, LDL-C and LDH levels did not differ among groups (Figures 1(g) and 1(h)). All rats received OGTT.  Figure 1(j) displays the serum glucose concentrations after an oral glucose challenge in these animals. It is apparent that serum glucose concentration in response to oral glucose was higher in the low and high STZ groups, which demonstrated that the diabetic model was successfully established.

### 3.2. Cardiac Remodelling in Different Experimental Groups

**Figure 2:**
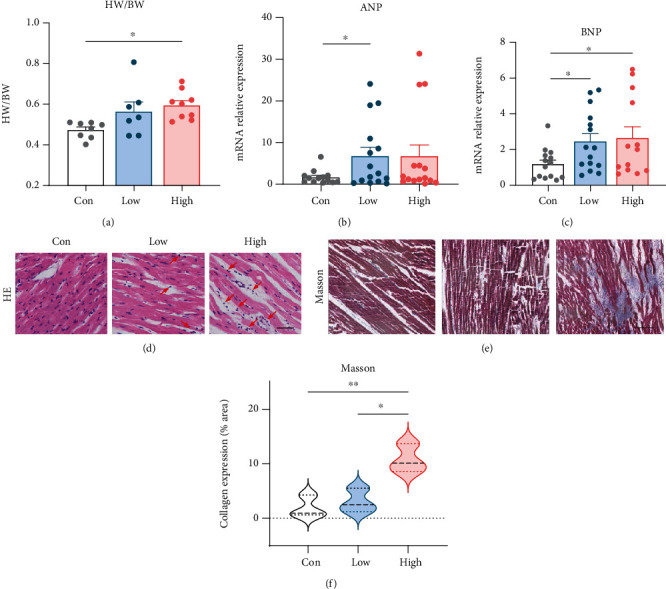
Cardiac remodeling in different experimental groups. (a) Heart weight normalized to body weight. qRT-PCR assay for *ANP* (b) and *BNP* (c) mRNA expression in myocardial tissue. Representative images of HE staining ((d), arrows indicated lymphocytic infiltration; scale bar, 12.5 *μ*m) and Masson's trichrome staining ((e): red, cardiomyocytes; blue, collagen fibers; scale bar, 50 *μ*m). (f) Quantitative analyses of the positive staining of collagen expression (results are from 3-4 images per group). ^∗^*P* < 0.05, ^∗∗^*P* < 0.01. Con: control group; Low: low STZ group; High: high STZ group; HW: heart weight; BW: body weight.

To determine the degree of cardiac remodelling in diabetic rats, cardiac weight index and myocardial collagen deposition were measured. As shown in Figure 2(a), heart weight was normalized to body weight and did not differ between the control and low STZ groups, while in the high STZ group the cardiac weight index was significantly enhanced relative to control. Increased ANP and BNP gene expression by qRT-PCR was observed in STZ groups when compared with controls (Figures 2(b) and 2(c)). HE staining (Figure 2(d)) revealed an approximately normal microstructure of cardiomyocytes in the control. In the high STZ group, cardiomyocytes showed interstitial edema and lymphocytic infiltration (marked with red arrow). These abnormal structures were less frequent in the low STZ group. Masson's trichrome staining demonstrated that interstitial collagen deposition was significantly increased in the high group as compared with the control group, but the low STZ group did not display increased collagen deposition in the myocardium (Figures 2(e) and 2(f)).

### 3.3. RNA-Seq Analysis Revealed a Differential Transcriptome in DM Rats

**Figure 3:**
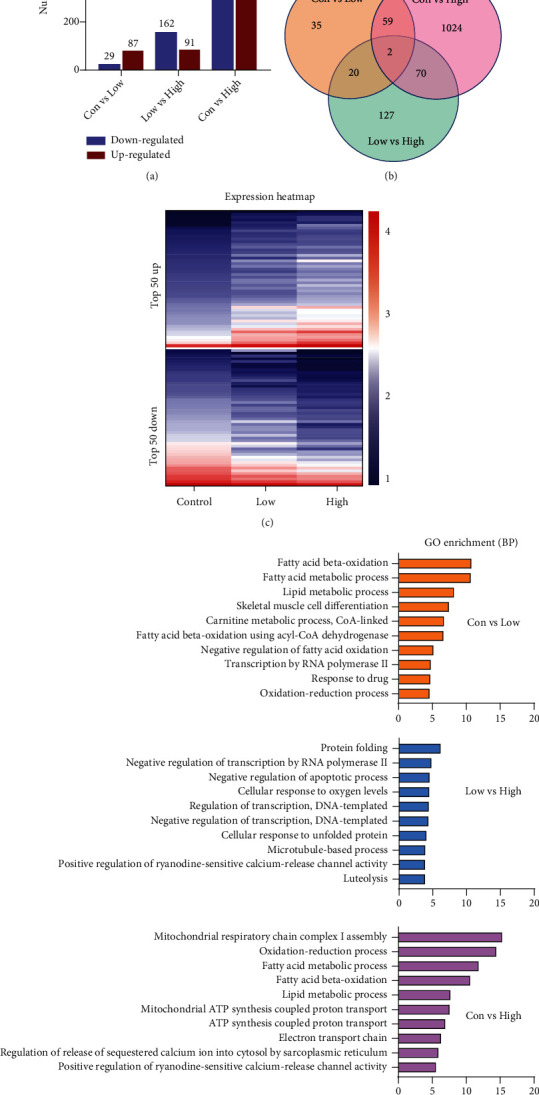
RNA-Seq analysis revealed differentially expressed genes (DEGs) in the left ventricle from control, low, and high doses of streptozotocin-injected high-fat diet rats. (a) The bar graph shows the number of up and downregulated DEGs in Con vs. high is great than Con vs. low and low vs. high comparison. (b) Venn diagram showing separate and overlapping expression of DEGs when the control, low, and high groups are compared with each other. Each circle represents a group of gene sets, and the areas superimposed by different circles represent the intersection of these gene sets. (c) Heatmap expression of the top 50 upregulated and 50 downregulated DEGs which selected from Con vs. high, and the reads value of these genes in the 3 groups are displayed. (d) The 10 most significantly (*P* < 0.05) enriched GO terms in the biological process branch are presented in three groups of comparisons. Con: control group; Low: low STZ treated group; High: high STZ treated group.

**Figure 4:**
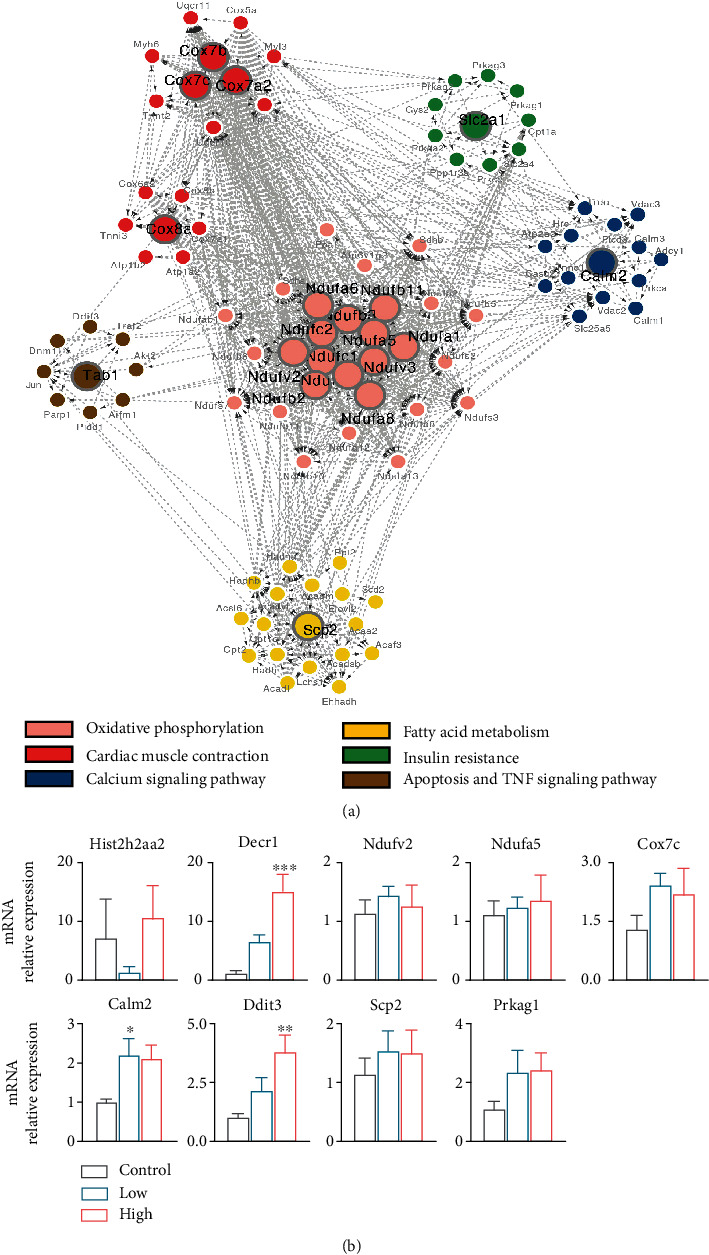
(a) Analysis of hub genes from protein-protein interaction (PPI) network for the candidate pathways. Nodes encircled in pink, red, blue, yellow, green, and brown indicate significant genes in pathways involved oxidative phosphorylation, cardiac muscle contraction, calcium signalling, fatty acid metabolism, insulin resistance, apoptosis, and TNF pathway, respectively. (b) Validation of key genes in ventricular tissue of rats in control, low and high STZ treatment groups using RT-PCR. ^∗^*P* < 0.05, ^∗∗^*P* < 0.01, ^∗∗∗^*P* < 0.001. Compared with control. *n* = 6 − 7 per group.

Previous studies have identified multiple intracellular pathways involved in the pathogenesis of diabetic cardiomyopathy [19], but the molecular cues underlying cardiac damage in DM remain elusive, especially in different diabetes status. To further investigate the molecular mechanisms behind the progression of diabetic damage, we performed genome-wide RNA-Seq by comparing control, low, and high STZ treated rats. We observed that 116 genes out of 37,246 total genes (87 upregulated and 29 downregulated) were differentially expressed in control vs. low STZ; 253 genes (91 upregulated and 162 downregulated) were differentially expressed in low vs. high STZ; 1189 genes (582 upregulated, and 607 downregulated) were differentially expressed in control vs. high (Figures 3(a) and 3(b)). Among these, there are 2 common differentially expressed genes (DEGs) from 3 comparison groups: *Hist2h2aa2*, histone cluster 2 H2A family member A2, which is the core component of the nucleosome, enables protein heterodimerization activity; *Decr1*, is an enzyme that participates in fatty acid *β*-oxidation and metabolism of polyunsaturated fatty enoyl-CoA esters^3^. These two common genes can be candidates to be further analyzed and validated as diagnostic markers to track the process of diabetic cardiomyopathy (Figure 4(b)). According to Log2-fold change between control and high STZ groups, the top 50 upregulated and 50 downregulated genes were selected, and the clean reads value of these genes in the 3 groups are displayed as a heatmap (Figure 3(c)), demonstrating the differential gene expression pattern between the 3 groups.

Based on the GO enrichment, the top 10 GO terms in biological processes which the DEGs were enriched according to the gene count are presented in Figure 3(d). GO analysis revealed that genes were enriched in “fatty acid beta-oxidation,” “fatty acid metabolism,” “lipid metabolic process,” and “skeletal muscle cell differentiation” from Con vs. Low; “protein folding,” “negative regulation of transcription by RNA polymerase II,” “negative regulation of apoptotic process,” and “cellular response to oxygen levels” by comparing low and high STZ; and “mitochondrial respiratory chain complex I assembly,” “oxidation-reduction process,” “fatty acid metabolic process,” and “fatty acid beta-oxidation” from control vs. high STZ. According to the KEGG pathway classification, we also selected key genes which related to fatty acid metabolism, oxidative phosphorylation, cardiac structure and function related, insulin resistance, calcium signalling, apoptosis, and TNF pathway (Figure S1) are significant changed between the control and high STZ groups. There is also a corresponding trend in the low STZ group. These results indicate a differential transcriptomic profile expressed in different stages of diabetic cardiomyopathy.

Dysregulation in a protein subnetwork may yield dysfunctional multiple protein subnetworks. We performed the protein-protein interaction (PPI) analysis to identify the functional subnetworks of the genes of interest for KEGG pathways using the web-based visualisation resource STRING. Nodes encircled in pink, red, blue, yellow, green, and brown indicate significant genes in pathways involved oxidative phosphorylation, cardiac muscle contraction, calcium signalling, fatty acid metabolism, insulin resistance, apoptosis, and TNF pathway, respectively (Figure 4(a)). All genes in this network were quantified and ranked by the clustering coefficient measurement with cytoscape software, in which the TOP 20 nodes were identified as hub genes (indicated in bigger icons) and listed at supplement table 2.

To validate the identified genes from control and STZ treated rats, myocardial tissues were extracted to measure mRNA levels. Two common DEGs from 3 comparison groups (*Hist2h2aa2* and *Decr1)* and 7 hub genes from listed supplement table 2 (*Ndufv2*, *Ndufa5*, *Cox7c*, *Calm2*, *Ddit3*, *Scp2*, and *Prkag1*, based on log_2_FC and *Q* values in RNA-Seq analysis) were selected. Only *Decr1*, *Calm2*, and *Ddit3* were expressed statistical significance when compare with control group (Figure 4(b)).

### 3.4. Measurements of Cardiac Oxidative Stress and Apoptosis in Different Experimental Groups

**Figure 5:**
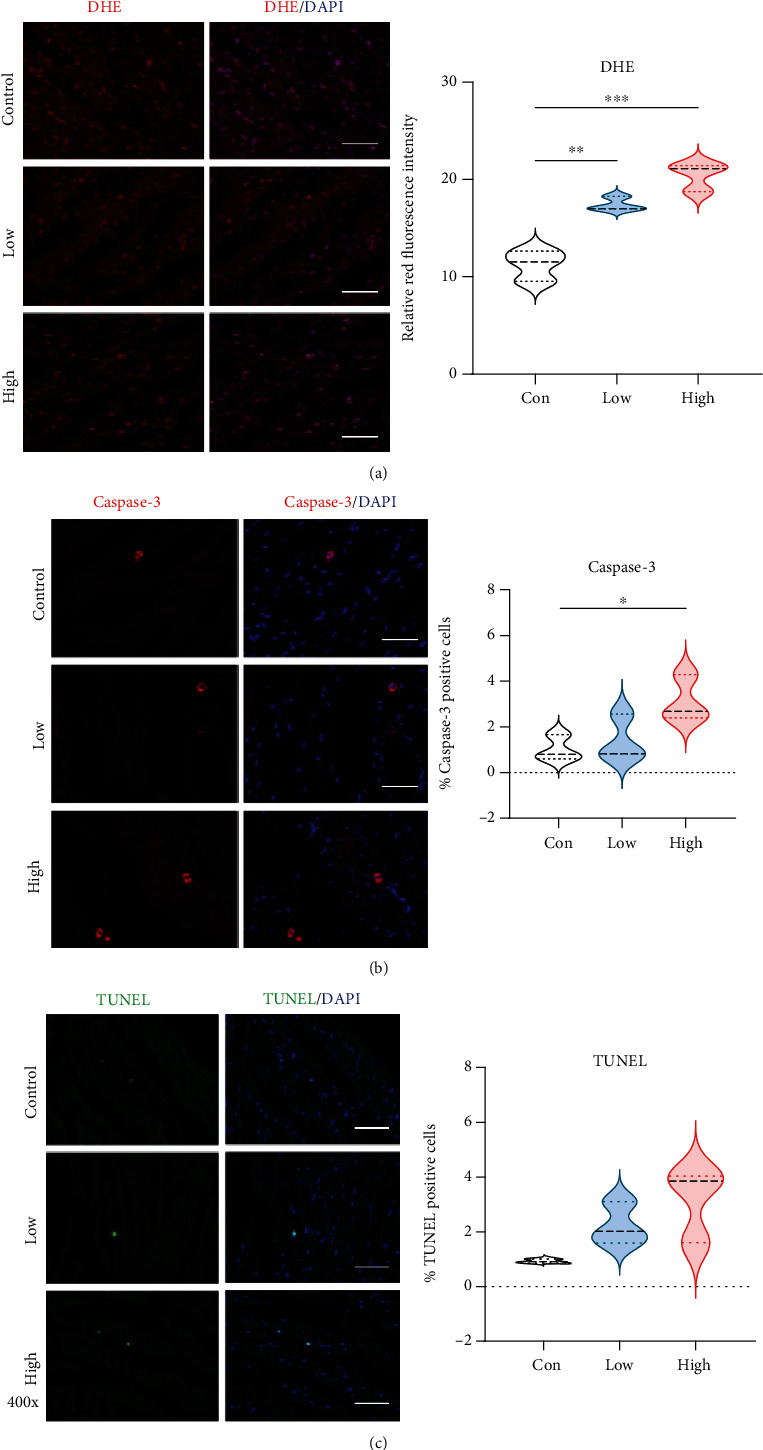
Cardiac oxidative stress and apoptosis in different experimental groups. (a) Representative images of DHE staining for myocardial tissues (red, DHE; blue, DAPI) and the average fluorescence intensity were summarized. (b) Representative images of immunofluorescence for apoptotic cells (red, caspase-3; blue, DAPI). The apoptotic index was quantified by Image-Pro Plus software. (c) Representative images of immunofluorescence for apoptotic cells (green, TUNEL; blue, DAPI). The apoptotic index was quantified by Image-Pro Plus software. Results are from 3-4 images per group. Data are expressed as mean ± SE. ^∗^*P* < 0.05, ^∗∗^*P* < 0.01, ^∗∗∗^*P* < 0.001. Con: control group; Low: low STZ treated group; High: high STZ treated group. Scale bar, 12.5 *μ*m.

Based on the results from RNA-Seq, we verified whether oxidative stress and apoptosis are altered in different stages of DCM rat models. ROS accumulation is known to play a major role in diabetic complications including diabetic cardiovascular disease. DHE staining showed that ROS production from the left ventricle trended upward in both low and high STZ groups and was significantly enhanced in the low and high STZ treated groups (Figure 5(a)).

To investigate cardiac apoptosis in diabetic hearts, caspase-3 immunofluorescence staining and TUNEL assay were performed. As shown in Figures 5(b) and 5(c), the high-dose group had a significant higher rate of myocardial apoptosis on caspase-3, but this did not reach a significant difference on TUNEL when compared with control. There was no significant difference between the control and low STZ groups.

### 3.5. Optical Mapping of Intracellular Calcium in Different Experimental Groups

**Figure 6:**
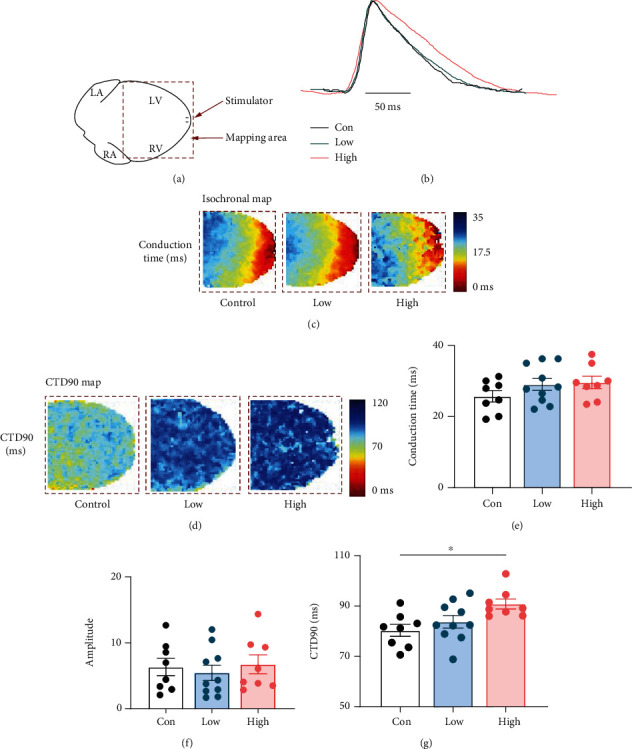
Calcium Rhod-2 optical mapping in isolated perfused hearts in different experimental groups. (a) Optical mapping configuration. (b) Comparison of typical calcium transients averaged over defined regions of interest. (c) Map of calcium transient conduction during 1 Hz stimulation. (d) Map of calcium transient duration at 90% recovery (CTD90) during 1 Hz stimulation. Bar charts display the average value of the calcium transient conduction time (e), amplitude (f), and CTD90 (g) changes of the control, low, and high STZ groups. Data are expressed as mean ± SE. ^∗^*P* < 0.05. Control: control group; Low: low STZ treated group; High: high STZ treated group.

From the RNA-seq analysis, we detected calcium signalling pathway genes, especially *Calm2* and *Atp2A2* that were significantly altered in the STZ treated group. To investigate whether the intracellular calcium transient was impaired in the pre and early stage of DCM rats, we performed optical fluorescence mapping using Rhod-2AM on sinus-paced Langendorff-perfused hearts (Figure 6(a)). The mapping of intracellular calcium transients at the anterior ventricular wall demonstrated that the conduction time (Figures 6(c) and 6(e)) and amplitude (Figure 6(f)) of the intracellular calcium transient did not significantly change with the intrinsic heart rate. However, there was a significant prolongation in the 90% calcium transient duration (CTD90, Figures 6(d) and 6(g)), especially in the high-dose group during high frequency (5 Hz) stimulation. The reduction of *Atp2A2* (ATPase Sarcoplasmic/Endoplasmic Reticulum Ca^2+^ Transporting 2) gene expression in high STZ group, which is responsible for calcium uptake into the sarcoplasmic reticulum (SR), may contribute to prolonging the CTD90.

### 3.6. Physiological Phenotyping of High Glucose-Treated Human iPSC-Derived Cardiomyocytes

#### 3.6.1. Cell Size, Glucose Uptake, ROS, and ATP Production in High Glucose-Treated iPSC-CM

**Figure 7:**
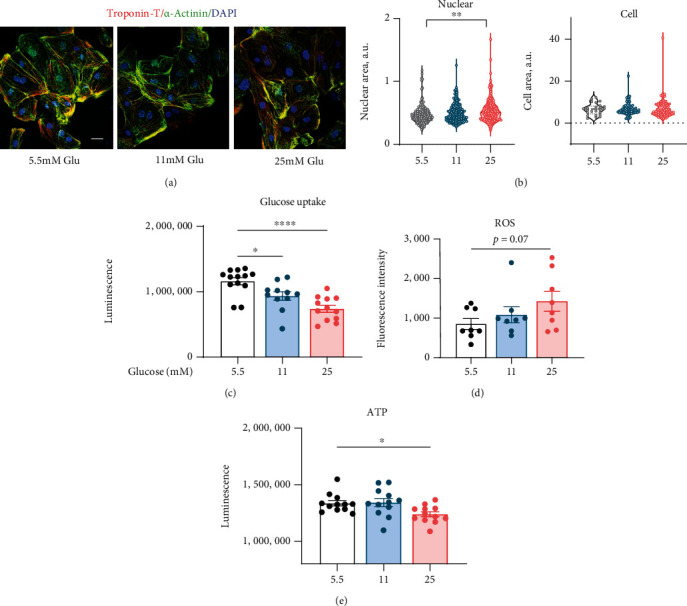
(a) iPSC-CMs stained positive for cardiac troponin T (cTnT, red), *α*-actinin (green), and DAPI (blue). Scale bar, 25 *μ*m. (b) Statistical comparison of nuclear area and cell area among different glucose treatment groups. Comparison of glucose uptake (c), reactive oxygen species (ROS, (d)), and ATP production (e) among different glucose treatment groups. Data are expressed as mean ± SE. ^∗^*P* < 0.05, ^∗∗^*P* < 0.01, ^∗∗∗∗^*P* < 0.0001.

Exposure to high levels of glucose increased both cell area and nuclear area (Figures 7(a) and 7(b)). iPSC-CMs of the 25 mmol/L glucose group presented a significant nuclear enlargement compared to 5.5 mmol/L control group, while the increase of nuclear size of the 11 mmol/L group was insignificant. Cell size also showed a tendency to increase with the elevation of glucose level, although the difference is less remarkable than nuclear change. Cellular and nuclear hypertrophies suggest activated cellular metabolic biosynthesis under the effect of high glucose. The glucose uptake (Figure 7(c)) and ATP production (Figure 7(e)) displayed significant suppressed; ROS production (Figure 7(d)) enhanced in the 25 mmol/L glucose group compared to 5.5 mmol/L control group. The glucose uptake was also reduced in the 11 mmol/L glucose group, but did not detect changes in ROS and ATP.

#### 3.6.2. [Ca^2+^]_i_ Measurement in hiPSC-CMs

**Figure 8:**
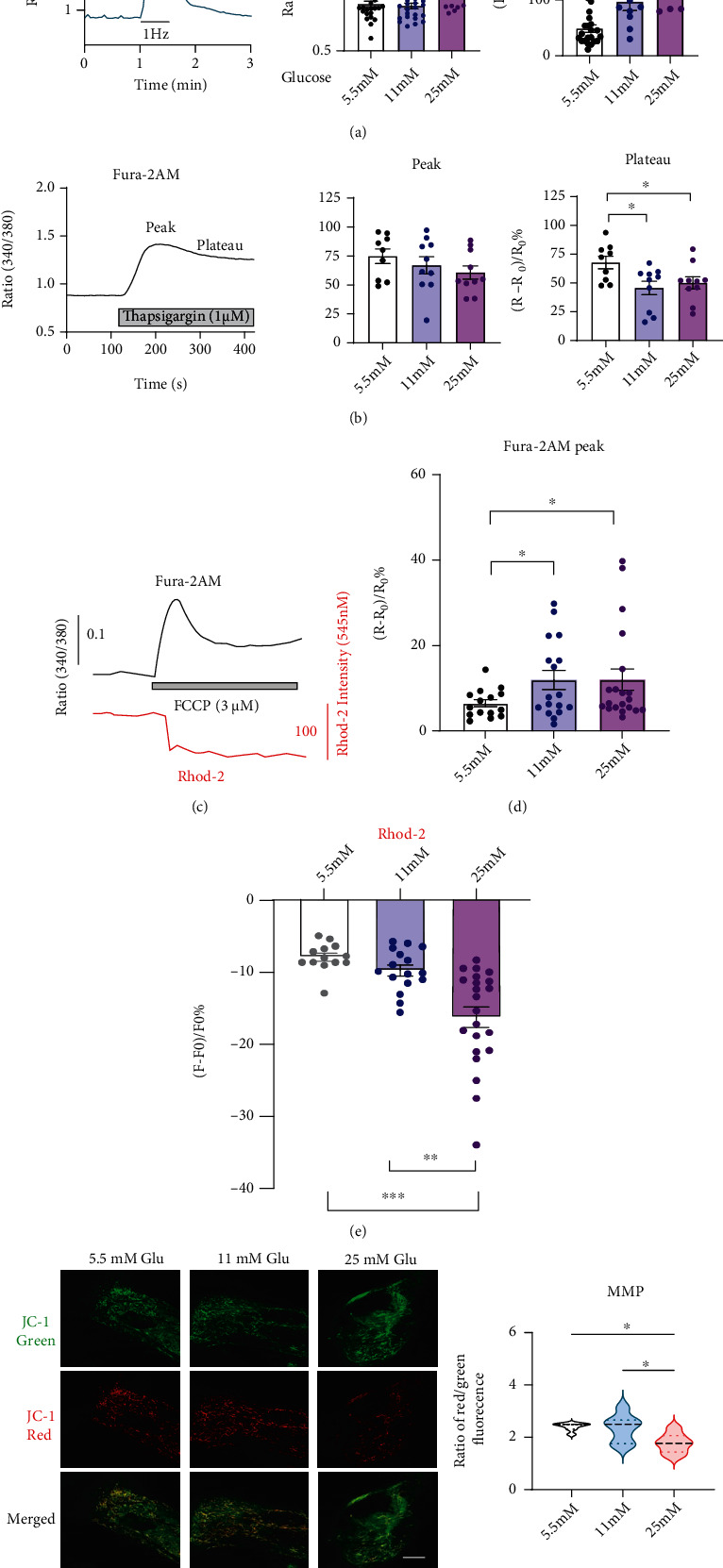
Functional phenotyping of high glucose-treated human iPSC-derived cardiomyocytes. (a) Fluorescence ratio profile showing the intracellular free calcium transients ([Ca^2+^]_i_) response to 1 Hz field stimulation using Fura-2AM in hiPSC-CMs (left). Measurement of baseline (middle) and peak (right) [Ca^2+^]_i_ in hiPSC-CMs with 5.5 mmol/L (control), 11 or 25 mmol/L glucose treatment. (b) [Ca^2+^]_i_ changes in hiPSC-CMs by inhibition of the sarcoplasmic reticulum Ca^2+^ pump with thapsigargin among the group. Peak and plateau were expressed as % of ratio changes. (c) Measurement of [Ca^2+^]_i_ with Fura-2AM (top, black line) and mitochondrial Ca^2+^ ([Ca^2+^]_m_) using Rhod-2/AM (bottom, red line) after mitochondrial Ca^2+^ store depletion with FCCP in hiPSC-CMs. (d) The peak evoked [Ca^2+^]_i_ after FCCP treatment among the groups. (e) The reduction of [Ca^2+^]_m_ changes after FCCP treatment among the groups of hiPSC-CMs. (f) Analysis of mitochondrial membrane potential (MMP) changes using the cationic JC-1 dye as a fluorescent probe in hiPSC-CMs. The changes were expressed as the ratio of red/green fluorescence intensity (results are analyzed from 6-8 images per group; scale bar, 10 *μ*m). Data are expressed as mean ± SE. ^∗^*P* < 0.05, ^∗∗^*P* < 0.01. ^∗∗∗^*P* < 0.001.

To explore if the high glucose environment could alter calcium handling in hiPSC-CMs, we first measured intracellular free calcium concentration ([Ca^2+^]_i_) using ratiometric recordings with Fura-2/AM. A trace for the calcium transient in response to 1 Hz field stimulation is shown in Figure 8(a). Baseline ratio was elevated in the 25 mmol/L glucose group, while no change was found from 11 mmol/L compared with control 5.5 mmol/L cells (Figure 8(b)). Stimulation evoked increases in [Ca^2+^]_i_ were significantly enhanced in both 11 and 25 mmol/L glucose groups when compared with 5.5 mmol/L control (Figure 8(c)), and the difference between the 11 and 25 mmol/L glucose groups was also remarkable. These results suggest that high glucose exposure impaired calcium handling in hiPSC-CMs.

Evoked [Ca^2+^]_i_ responses are influenced by multiple factors. To check if the sarco(endo)plasmic reticulum calcium transport ATPase (SERCA) pump, which is modulated by the protein products of the *Atp2a2* gene, a SERCA pump inhibitor thapsigargin was introduced into the cells (Figure 8(b)). 1 *μ*mol/L thapsigargin showed rapidly increased [Ca^2+^]_i_. This enhancement tended to reduce with the elevation of glucose level on both peak and plateau measurements, although the difference is less remarkable at the peak, indicating that impairment of SERCA pump may be involved in the mechanism of enhanced calcium handling in high glucose treated hiPSC-CMs.

#### 3.6.3. Mitochondrial [Ca^2+^] and Mitochondrial Membrane Potential (MMP) Level in hiPSC-CMs

We applied the proton uncoupler FCCP (3 *μ*mol/L) to depolarize the inner mitochondrial membrane, using Fura2-AM, to monitor the release of stored mitochondrial Ca^2+^ (Figures 8(c) and 8(d)) or directly measure mitochondrial Ca^2+^ content using the Rhod-2/AM (5 *μ*mol/L) which can selectively accumulate within mitochondria (Figures 8(c) and 8(e)). The peak of the [Ca^2+^]_i_ transient increased in the hiPSC-CMs with 11 or 25 mmol/L glucose group when compared with 5.5 mmol/L (control) group, but there was no significant difference between high glucose groups (Figure 8(d)). For [Ca^2+^]_m_ (Figure 8(e)), FCCP significantly reduced the mitochondrial Ca^2+^ signal in the 25 mmol/L glucose group when compared with the control, but there was minimal difference between 11 mmol/L and control groups. Interestingly, there was a significant reduction in 25 mmol/L in comparison with the 11 mmol/L group. Altogether, these data indicate that mitochondria play an important role in the modulation of Ca^2+^ signalling resulting from a high glucose environment.

To exclude a hyperosmolar effect, we cultured iPSC-CMs in 5.5 mmol/L glucose plus 19.5 mmol/L mannitol (high mannitol group) to achieve the same osmolarity as 25 mmol/L glucose medium. We found no difference in baseline calcium transient ratios between control (5.5 mmol/L glucose, 0.959 ± 0.008, *n* = 15) and the high mannitol group (0.935 ± 0.016, *n* = 9, Figure S2A). After applied FCCP, there was no significant difference in the peak intracellular calcium transient between the two groups (6.496 ± 0.183% vs. 8.004 ± 0.231%, Figure S2B). These results suggest that hyperosmolarity of our culture conditions does not affect intracellular calcium transients in iPSC-CMs.

We used both fluorescence microscopy and quantitative fluorescence measurements to evaluate the MMP changes in 5.5, 11, or 25 mmol/L glucose-treated hiPSC-CMs (Figure 8(f)). We observed a significant reduction in mitochondrial membrane polarization as indicated by an increase in the MitoProbe JC-1 red:green fluorescence ratio in 25 mmol/L glucose-treated hiPSC-CMs, however, we did not observe any changes in the 11 mmol/L glucose group, suggesting that high glucose affects the MMP. We also performed transmission electron microscopy (TEM) to examine whether there were differences in the ultrastructural features between 5.5 and 25 mmol/L glucose-treated hiPSC-CM. Myofibrils were well organized into sarcomeres with clearly aligned Z-lines in both groups, whereas mitochondria and endoplasmic reticulum (ER) had no morphological changes (Figure S3).

## 4. Discussion

In this study, we have successfully established different stages of DM in a rat model by feeding a high-fat diet combined with different doses of STZ to emulate pre- and early DCM status. We provide several lines of evidence demonstrating functional and structural alterations in the myocardium because of metabolic and cellular abnormalities in the different stages of DM. We showed that Ca^2+^ signalling homeostasis, ROS generation, and apoptotic cell death regulation are dysregulated in the early stage of DCM. We used RNA-Seq to explore a differential transcriptomic profile and captured the effect of changes in gene expression and important signalling pathways at different stages of DM. Moreover, we clarified further underlying molecule correlates by using high glucose-treated iPSC-CM that mimic a diabetogenic environment.

T2DM is characterized by a metabolic disorder that elevates blood glucose concentration. It is well known that chronic hyperglycemia has been associated with many aspects of health in T2DM patients, including severe cardiovascular outcomes. The typical clinical features of cardiac dysfunction associated with T2DM include reduced ventricular compliance and diastolic function, decreased exercise capacity, as well as increased incidence of cardiac arrhythmias [20]. T2DM patients are identified as a high-risk subgroup for developing HF. In addition, impaired glucose tolerance (IGT) has been shown to contribute to poor prognosis and is an independent factor in patients with HF [21]. Although diabetic cardiac dysfunction has been recognized for many years through a broad range of investigations, effective strategies for cardiac prevention and treatment remain elusive.

Accumulating evidence demonstrated that high blood glucose levels are strongly associated with a greater risk of cardiac abnormality among T2DM. Patients with poor glycemic control have an increased incidence of cardiovascular disease and experience worse clinical outcomes [19]. Reaven et al. reported that participants with T2DM who had intensive glucose control had a lower risk of cardiovascular events, while there was no evidence of a mortality benefit with intensive glucose control [22]. Furthermore, some studies confirmed that there were no substantially beneficial effects of intensive glycemic control on major cardiovascular outcomes and all-cause mortality in T2DM patients [23]. These findings suggest that the contribution of intensive glucose control in the reduction of major cardiovascular outcomes remains an open question.

The onset of cardiac injury occurs early and before the onset of measurable cardiac dysfunction. More importantly, effective approaches that can reverse cardiac injury are limited. The effects of different blood glucose levels on cardiac injury, and mechanisms underlying such effects are not fully elucidated. Early detection of the effects of T2DM on the heart would enable the optimal implementation of effective therapies that prevent HF development. Therefore, the purpose of the present study was to characterize the underlying cardiac molecular mechanisms and cardiac abnormalities in T2DM rats with different diabetes status, which may provide evidence for optimal timing and strategies of prevention and treatment.

In this study, we developed T2DM animal model by high-fat diet combined with STZ injection, as previously described [9]. The low dose group received a single intraperitoneal injection of STZ at 35 mg/kg, while the high-dose group received a single intraperitoneal injection of STZ at 50 mg/kg. Various investigators have used protocols ranging from a single dose of STZ at 50 to 150 mg/kg [24, 25]. We adopted a relatively low-dose strategy, because this strategy minimizes the nonspecific toxic effects of high-dose STZ [14]. After 9 weeks, our results from the present study showed that rats from the high-dose group exhibited significant higher levels of fasting glucose, insulin, TC, TG, FFA, and HOMA-IR compared with the low dose group, while the insulin, TC, TG, FFA, and HOMA-IR levels did not differ between the low dose group and the control group. Furthermore, the rats from the low dose group received OGTT, which confirmed the success of T2DM establishment. It has been reported that hyperglycemia is associated with an altered myocardial substrate, which has been hypothesized to lead to cardiac injury [26].

Interstitial fibrosis is important mechanism underlying the pathogenesis of cardiac injury, which could aggravate myocardial stiffness. The degree of cardiac remodelling in the two T2DM rat models was assessed. In the high STZ treated group, the cardiac weight index and myocardial collagen deposition were significantly increased when compared with controls. In addition, by RNA-Seq, we also detected cardiac structure modulated genes such as *Myh6*, *Tnnt2*, *Tpm1*, *Myl3*, *Tnni3*, *Ttn*, and *Tnnc1* were reduced, *Tab1* (TGF-beta activated kinase 1 and MAP3K7-binding protein 1) which participates fibrotic response was enhanced in the high STZ dose group (Figure S1). However, these indicators did not find any statistical differences in the low STZ treated group. Combining the results of basic parameters, we confirmed that our high and low doses of STZ treated T2DM models belong to pre and early stage of DCM. We also found the *ANP* and *BNP* mRNA expression of the ventricular tissues enhanced in STZ-treated groups relative to control (Figures 2(b) and 2(c)). Several lines of evidence have indicated that BNP elevation represents the earliest change of cardiac injury, preceding the appearance of cardiac remodelling [27]. This is consistent with our findings and further confirmed cardiac hypertrophy in our T2DM model.

Oxidative stress is one of the major components triggering cardiac changes in DM. The increase in ROS production may subsequently induce apoptosis, inflammation, and other pathologic changes [28, 29]. The present study showed that ROS production was enhanced in both low and high-dose group and was much higher in the high-dose group (Figure 5(a)). The high-dose STZ group had a higher rate of myocardial apoptosis with caspase-3 activity (Figure 5(b)). Moreover, our RNA-Seq results confirmed that the fatty acid metabolism, oxidative phosphorylation, apoptosis, and TNF pathway-related genes were altered in the high-dose group. These results indicate that the accumulation of ROS occurs from the pre to the early onset of DCM. Our work agrees with the previous report that excess generation of ROS is considered a central mechanism for T2DM-related cardiac apoptosis and remodelling during both the early and late stages of diabetic cardiomyopathy [30]. We also detected two glucose transporters, *Slc2a4* (Glut4) and *Slc2A1* (Glut1) genes decreased in the high-dose STZ group (Figure S1). This induced impairment of glucose metabolism, while increased fatty acid metabolism led to mitochondrial dysfunction, which together increase ROS production [31]. We validated some key genes by real-time PCR and found *Decr1* (an enzyme which participates in fatty acid *β*-oxidation), *Calm2* (associated with cardiac arrhythmias), and *Ddit3* (a proapoptotic transcription factor) expressed a statistically significant increase when compare with the control group. These genes might be therapeutic targets for the treatment of diabetic cardiomyopathy in the future.

The prevalence and severity of T2DM-induced cardiac injury warrant a deeper investigation of the mechanisms and implicating factors. In recent years, the emergence of iPSC has allowed for the generation of iPSC-CMs, human-derived cells that can be applied to the in vitro study [32]. In the present study, iPSC-CMs were cultured in physiological (5.5 mmol/L glucose) or high glucose environment (11 and 25 mmol/L glucose) conditions. We demonstrated cellular and nuclear hypertrophy in the 25 mmol/L glucose-treated iPSC-CM, and the glucose uptake, ROS, and ATP production were also significantly altered. These findings were consistent with our DM rat model results. In addition, mitochondrial membrane potential was significantly decreased in the 25 mmol/L group, which indicated impaired electron transport and oxidative phosphorylation. Evoked [Ca^2+^]_i_ responses are influenced by multiple factors, including Ca^2+^ entry, extrusion across the plasma membrane, Ca^2+^ uptake and release from internal stores, and endogenous and exogenous Ca^2+^ buffering [33, 34]. The present study suggests a central role for mitochondria in impaired calcium transients, alongside reduced SERCA pump activity resulting in slowing of SR Ca^2+^ reuptake, which could contribute to high cytosolic calcium, as well as prolonged intracellular calcium transient duration (CTD90) measured by optical mapping in the high STZ treated DM rats. Calcium transportation is a fundamental biological process that has critical effects on cellular metabolism, signalling, and survival [35].

**Figure 9:**
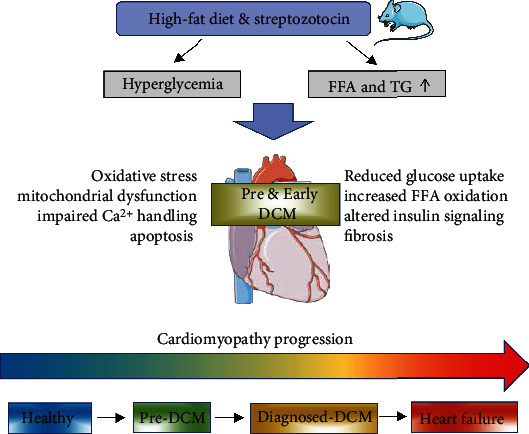
Summary diagram illustrating the potential mechanism of pre and early state of diabetic cardiomyopathy during cardiomyopathy progression in a diabetic rat model induced by high-fat diet combined with Streptozotocin injection.

In conclusion, cardiac remodelling, fibrosis, increased stiffness, and cardiomyocyte loss are all consequences of dysregulated glucose and lipid metabolism that triggers oxidative stress as well as mitochondrial dysfunction during the development of DCM (Figure 9). In this study, we have profiled the different stages of T2DM in a rat model by feeding high-fat diet combined with difference doses of STZ to emulate pre and early DCM status. Moreover, high glucose-treated iPSC-CM mimics a diabetogenic environment. This experimental framework may be utilised to investigate pathways underlying the progression of DCM.

## Data Availability

The data used to support the findings of this study are available from the corresponding authors upon reasonable request.

## Supplementary Materials

Supplementary Materials Supplementary table 1: list of primer sequences used for RT-PCR analysis. Supplementary table 2: hub genes from PPI network for the candidate pathways. Figure S1: bar graph to compare the TPM (transcript per million) values of key genes from RNA-Seq analysis according to the KEGG pathway classification. List of DEGs selected from the comparison of control vs. high STZ treated group, which related to fatty acid metabolism (a), oxidative phosphorylation (b), calcium signalling (c), cardiac structure and function related (d), insulin resistance (e), and apoptosis and TNF pathway (f). Figure S2: intracellular free calcium concentration on the baseline (a) and after treatment with 3 *μ*mol/L FCCP (b) in hiPSC-CMs exposed to 5.5 mmol/L glucose or high mannitol (5.5 mmol/L glucose with 19.5 mmol/L mannitol) media. Figure S3: transmission electron microscopy (TEM) of human induced pluripotent stem cell-derived cardiomyocytes (iPSC-CMs) treated in 5.5 (a) and 25 mmol/L glucose (b) culture media. ER: endoplasmic reticulum. Scale bar: 2 *μ*m.
