# Supplementary material for: Molecular Correlates of Early Onset of Diabetic Cardiomyopathy: Possible Therapeutic Targets
Source: Oxid Med Cell Longev. 2022 Apr 14;2022:9014155. doi: 10.1155/2022/9014155 (PMC9023181; doi:10.1155/2022/9014155)
Supplement: Supplementary Materials — Supplementary table 1: list of primer sequences used for RT-PCR analysis. Supplementary table 2: hub genes from PPI network for the candidate pathways. Figure S1: bar graph to compare the TPM (transcript per million) values of key genes from RNA-Seq analysis according to the KEGG pathway classification. List of DEGs selected from the comparison of control vs. high STZ treated group, which related to fatty acid metabolism (a), oxidative phosphorylation (b), calcium signalling (c), cardiac structure and function related (d), insulin resistance (e), and apoptosis and TNF pathway (f). Figure S2: intracellular free calcium concentration on the baseline (a) and after treatment with 3 μmol/L FCCP (b) in hiPSC-CMs exposed to 5.5 mmol/L glucose or high mannitol (5.5 mmol/L glucose with 19.5 mmol/L mannitol) media. Figure S3: transmission electron microscopy (TEM) of human induced pluripotent stem cell-derived cardiomyocytes (iPSC-CMs) treated in 5.5 (a) and 25 mmol/L glucose (b) culture media. ER: endoplasmic reticulum. Scale bar: 2 μm. [file 9014155.f1.pdf]

MS ID: 9014155

Online Supplement:

Molecular correlates of early onset of diabetic cardiomyopathy: possible therapeutic targets

Dongjuan Wang*[1], Kun Liu*[2], Jinyan Zhong[1], Xin Li[3], Jie Zhang[3], Gongxin Wang[4], Ni Li[2], Tianwen Li[5], Harvey Davis[6], Ibrahim El-gaby[2], Guoliang Hao[2,4], Honghua Ye[†1], Dan Li[†2]

1. Department of Cardiology, HwaMei Hospital, University of Chinese Academy of Sciences, Ningbo, Zhejiang, 315000, China
2. Burdon Sanderson Cardiac Science Centre and BHF Centre of Research Excellence, Department of Physiology, Anatomy and Genetics, University of Oxford, Oxford, OX1 3PT, UK
3. Department of Endocrinology, HwaMei Hospital, University of Chinese Academy of Sciences, Ningbo, Zhejiang, 315000, China
4. Henan SCOPE Research Institute of Electrophysiology Co. Ltd., Kaifeng 475000, China.
5. Ningbo Institute of Life and Health Industry, University of Chinese Academy of Sciences, Ningbo, Zhejiang, 315000, China
6. Department of Neuroscience, Physiology and Pharmacology, University College London, London WC1E 6BT, UK

* These authors contributed equally to this work

† ***Correspondences:***
Dan Li
E-mail: dan.li@dpag.ox.ac.uk

Honghua Ye
E-mail: lindayenbzjch@163.com

Supplementary Methods

Transmission Electron Microscopy: Human induced pluripotent stem cell-derived cardiomyocytes (iPSC-CMs) were processed as a monolayer on 12 mm glass coverslips. Samples were fixed with 2.5% glutaraldehyde + 2% PFA in 0.1M PIPES buffer, pH 7.4, pre-warmed to 37°C for 1hr at room temperature and then overnight 4°C. Samples were washed with 0.1M PIPEs buffer, post-fixed with 1% OsO4 +1.5% potassium ferrocyanide in 0.1M PIPES buffer, washed extensively with water and then stained with 0.5% uranyl acetate overnight at 4°C. Samples were then dehydrated using an ethanol series (30%, 50%, 70%, 80%, 90%, 95%) for 10 min at each step, then transferred to 100% ethanol for 60 mins with 3 solution changes. Samples were then infiltrated with TAAB Low Viscosity resin as follows: 25% resin in ethanol for 1 hr, 50% resin in ethanol for 2 hrs, 75% resin in ethanol for 1hr then 100% resin overnight. Samples were then incubated in 100% resin for a further 8 hrs with two fresh changes of resin, then embedded by inverting the coverslip cell side down onto a BEEM capsule tube filled with fresh resin and polymerised at 60°C for 48 hrs. Coverslips were snapped off the polymerised blocks using liquid nitrogen, leaving the cell monolayer embedded on the top surface of the block. Ultrathin sections (90nm) were obtained using a Diatome diamond knife on a Leica EM UC7 ultramicrotome and were transferred to 300 mesh copper grids. Sections were post-stained with lead citrate for 5 mins, washed with water and air dried, then imaged in a FEI Tecnai 12 TEM operated at 120kV using a Gatan OneView camera.

Supplementary table 1: List of primer sequences used for RT-PCR analysis.

Gene	Primer sequence（5′-3′）	
	Forward	Reverse
ANP	GCAAACATCAGATCGTGCCC	GGTGGTCTAGCAGGTTCTTGAAA
BNP	ACAATCCACGATGCAGAAGCTG	TCTGCCCAAAGCAGCTTGAA
GAPDH	GCAAGTTCAACGGCACAG	CGCCAGTAGACTCCACGAC
Hist2h2aa2	CGGACAATCGGGTGTCTGTC	CATCACGAGAGCGCAAACAG
Decr1	TGTGATCGCGAGCAGGAATA	GCCCTGCAACTTTGATCAGC
Ndufv2	TGGAGAGACTACGCCTGACA	TGGGTGTCAGATCCTCGTAG
Ndufa5	GGAGCAGCTTCTACGTTCGAT	CACCAGGCCAGTAGTCTTCTTC
Cox7c	CTCGTTCCGGATAGCAAGGT	AACGGCAAATTCTTCCCCG
Calm2	GAGCGAGTCGAGTGGTTGTC	AGTCAGTTGGTCAGCCATGC
Ddit3	ACCTGAGGAGAGAGTGTTCCA	GGACACTGTCTCAAAGGCGA
Scp2	ACGTGAAGAAC GGCAAAGGA	CCTTGAAAGAAGGCCGACTG
Prkag1	GTGCTAGCAATGGAGTCGGT	TCGATTCCGGGGTCTCTTGA

Supplementary table 2: Hub genes from PPI network for the candidate pathways.

Gene Symbol	Chromo-some	Strand	Average TPM			log_2FC	Q value	Regulation	KEGG pathway
			Control	Low	High	High / Control			
Ndufa1	chrX	-	646.93	559.15	568.21	-0.3101	0.0160	down	Oxidative phosphorylation
Ndufa5	chr4	-	1081.98	971.80	934.31	-0.3339	0.0008	down	Oxidative phosphorylation
Ndufa6	chr7	-	949.12	816.20	847.06	-0.2845	0.0013	down	Oxidative phosphorylation
Ndufa8	chr3	-	664.26	577.72	608.01	-0.2492	0.0273	down	Oxidative phosphorylation
Ndufb11	chrX	+	907.51	773.86	814.09	-0.2755	0.0120	down	Oxidative phosphorylation
Ndufb2	chr4	+	564.21	475.21	484.49	-0.3418	0.0468	down	Oxidative phosphorylation
Ndufb3	chr9	+	729.33	603.66	634.78	-0.3224	0.0038	down	Oxidative phosphorylation
Ndufb4	chr11	+	1624.31	1393.59	1498.30	-0.2383	0.0469	down	Oxidative phosphorylation
Ndufc1	chr2	-	317.38	267.58	279.25	-0.3078	0.0058	down	Oxidative phosphorylation
Ndufc2	chr1	+	493.73	420.99	440.70	-0.2819	0.0386	down	Oxidative phosphorylation
Ndufv2	chr9	-	1411.97	1172.10	1248.56	-0.2975	<0.0001	down	Oxidative phosphorylation
Ndufv3	chr20	+	874.49	813.05	737.96	-0.3272	0.0392	down	Oxidative phosphorylation
Cox7a2	chr8	-	2081.70	1732.82	1890.74	-0.2601	0.0147	down	Cardiac muscle contraction/ Oxidative phosphorylation
Cox7b	chrX	+	2974.63	2554.19	2653.78	-0.2862	0.0234	down	Cardiac muscle contraction/ Oxidative phosphorylation
Cox7c	chr2	-	1533.15	1265.23	1342.19	-0.3147	0.0161	down	Cardiac muscle contraction/ Oxidative phosphorylation
Cox8a	chr1	-	405.77	525.89	540.25	0.2923	<0.0001	up	Cardiac muscle contraction/ Oxidative phosphorylation
Calm2	chr6	-	197.90	239.12	156.31	-0.4607	0.0029	down	Calcium signaling pathway
Scp2	chr5	-	313.81	255.55	278.74	-0.2939	0.0085	down	Fatty acid metabolism
Slc2a1	chr5	+	16.73	21.40	7.94	-1.2035	0.0007	down	Insulin resistance
Tab1	chr7	+	9.10	9.18		0.3858	0.0311	up	Apoptosis and TNF signaling pathway
					Substitutions*				
Prkag1	chr7	-	92.01	83.36	84.95	-0.2336	0.0431	down	Insulin resistance
Ddit3	chr7	+	354.54	523.16	582.45	0.6035	0.0041	up	Apoptosis and TNF signaling pathway

* Considering the low abundance of Slc2a1 and Tab1, the highly enriched Prkag1 and Ddit3 were selected as candidates to ensure the presence of plausible hub genes in the Insulin resistance pathway and Apoptosis and TNF signalling pathway.

Figure S1

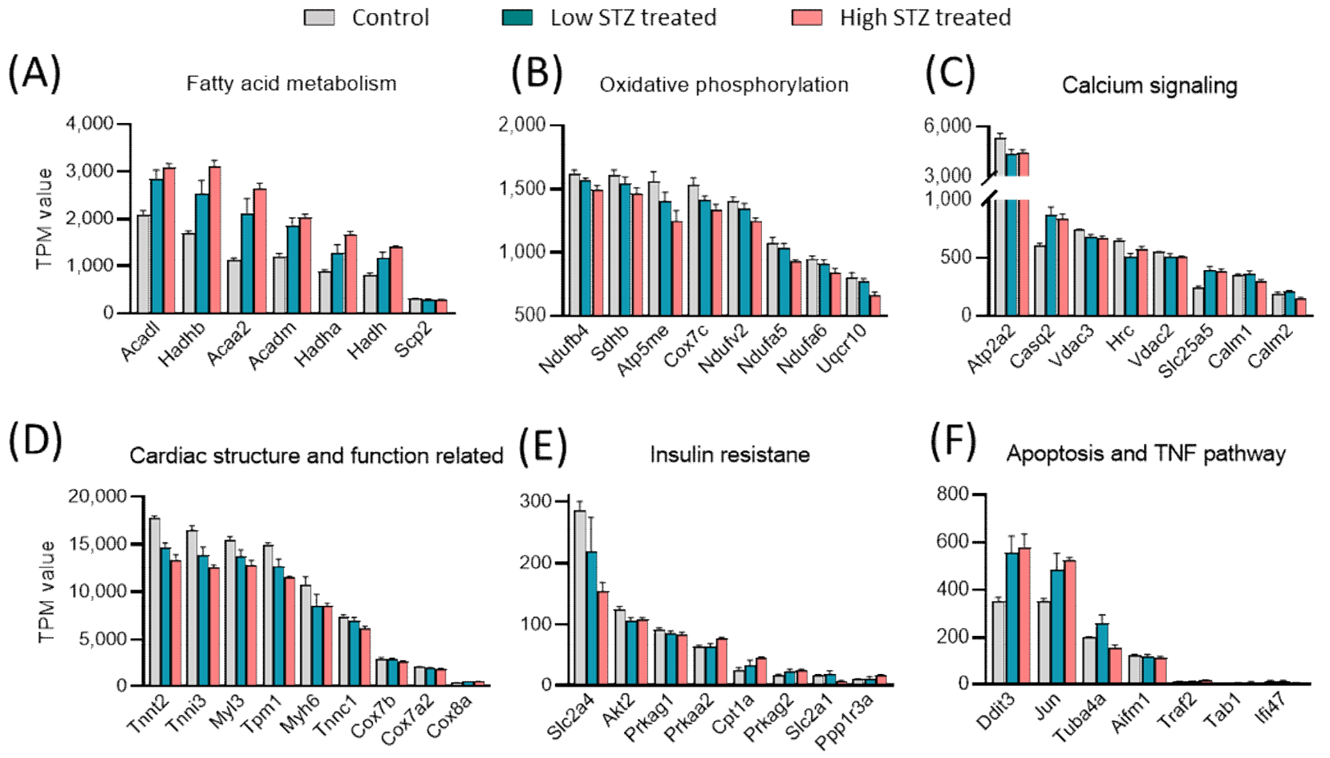

Figure S1: Bar graph to compare the TPM (transcript per million) values of key genes from RNA-Seq analysis according to the KEGG pathway classification. List of DEGs selected from the comparison of Control vs High STZ treated group, which related to fatty acid metabolism (**A**), oxidative phosphorylation (**B**), calcium signalling (**C**), cardiac structure and function related (**D**), Insulin resistance (**E**) and apoptosis and TNF pathway (**F**).

Figure S2

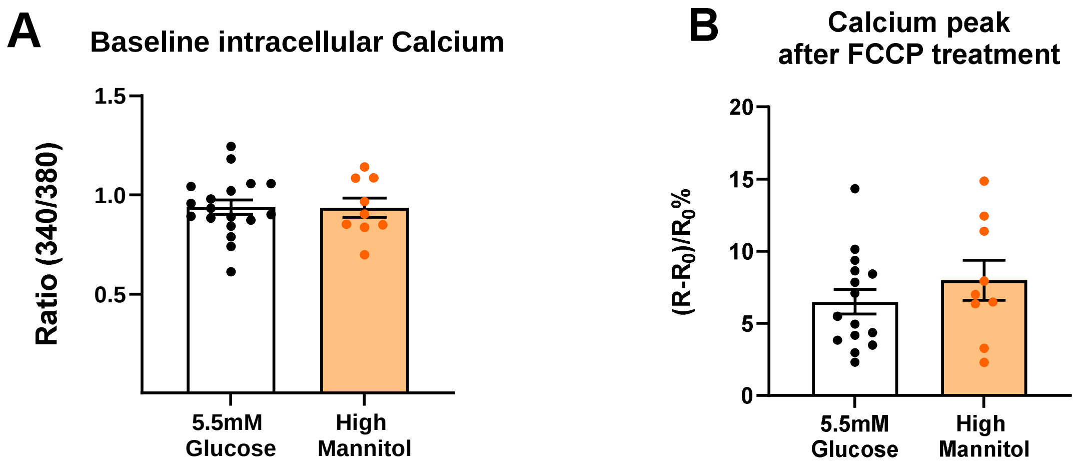

Figure S2: Intracellular free calcium concentration on the baseline (**A**) and after treatment with 3 μmol/L FCCP (**B**) in hiPSC-CMs exposed to 5.5 mmol/L Glucose or high Mannitol (5.5 mmol/L glucose with 19.5 mmol/L Mannitol) media.

Figure S3

A. **5.5mmol/L Glucose**

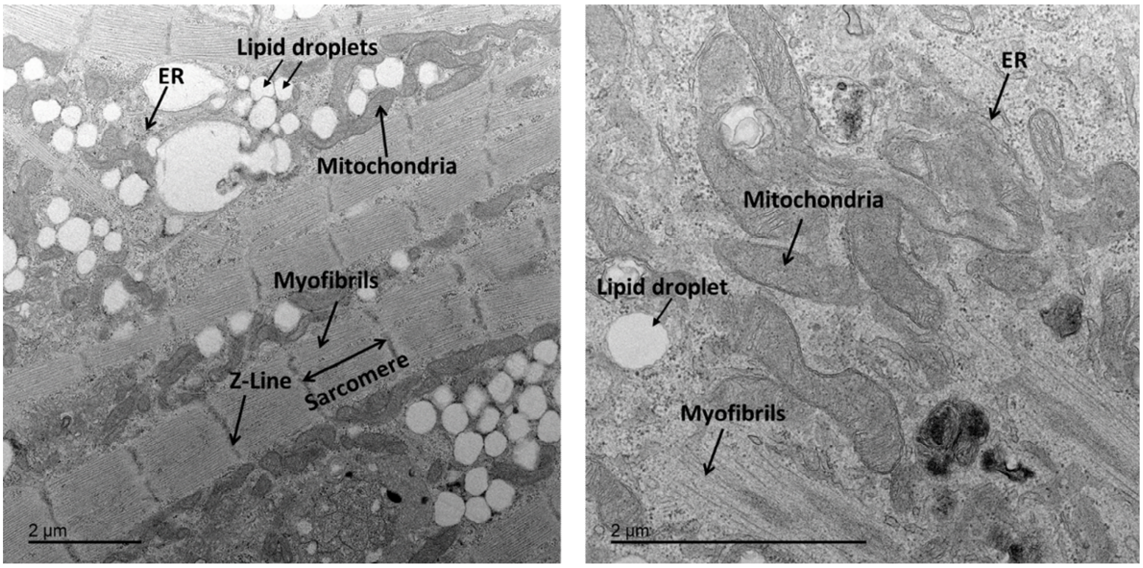

B. **25mmol/L Glucose**

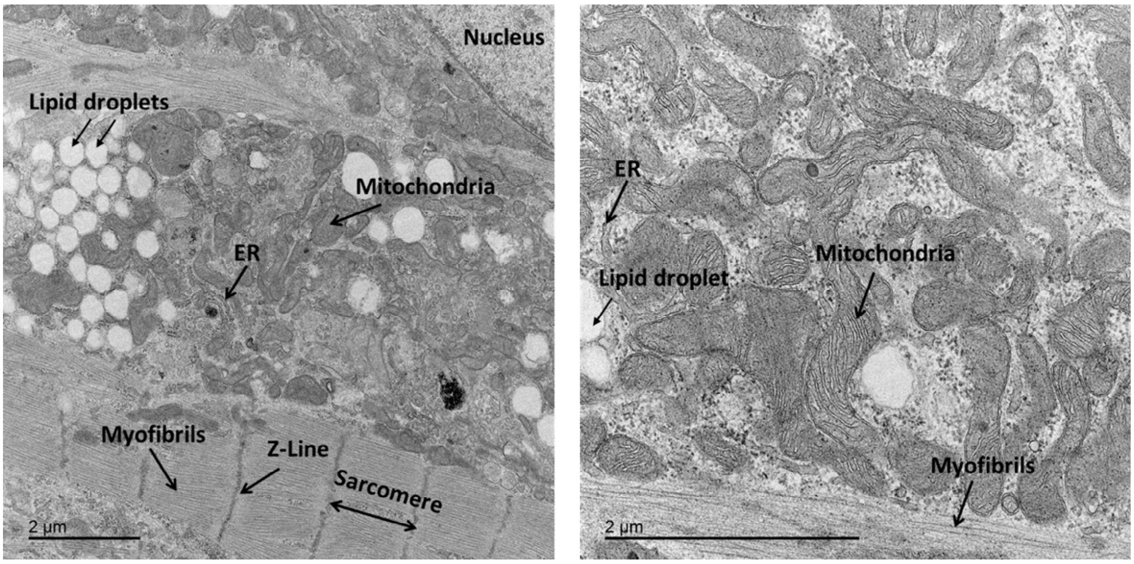

Figure S3: Transmission electron microscopy (TEM) of human induced pluripotent stem cell-derived cardiomyocytes (iPSC-CMs) treated in 5.5 (**A**) and 25 mmol/L glucose (**B**) culture media. ER: Endoplasmic reticulum. Scale bar: 2 µm.
